# Harnessing *Streptomyces* for the Management of Clubroot Disease of Chinese Cabbage (*Brassica rapa* subsp. *Pekinensis*)

**DOI:** 10.3390/plants14142195

**Published:** 2025-07-16

**Authors:** Shan Chen, Yang Zheng, Qing Wang, Rong Mu, Xianchao Sun, Guanhua Ma, Liezhao Liu, Jiequn Ren, Kuo Huang, Guokang Chen

**Affiliations:** 1 Chongqing Key Laboratory of Plant Disease Biology, College of Plant Protection, Southwest University, Chongqing 400716, China; chen15807436519@163.com (S.C.); 13656607423@163.com (Q.W.); murong3430@163.com (R.M.); sunxianchao@163.com (X.S.); nikemgh@swu.edu.cn (G.M.); 2 Institute of Vegetable and Flower Research, Chongqing Academy of Agricultural Sciences, Chongqing 400055, China; 3 Zhejiang Jiashan Forestry Technology Extension Station, Jiashan 314199, China; 4 College of Agronomy and Biotechnology, Academy of Agricultural Sciences, Southwest University, Chongqing 400715, China; liezhao@swu.edu.cn; 5 The Chongqing Three Gorges Academy of Agricultural Sciences, Wanzhou 404155, China; renjiequn@outlook.com; 6 College of Food Science and Technology, Huazhong Agricultural University, Wuhan 430070, China

**Keywords:** Chinese cabbage, biocontrol, clubroot, *Streptomyces*

## Abstract

Clubroot, caused by *Plasmodiophora brassicae* Woronin, poses a major threat to Chinese cabbage (*Brassica rapa* subsp. *pekinensis*) production worldwide, significantly impacting crop yield, quality, and economic value. Biological control represents a promising approach since it is non-toxic and eco-friendly, and it reduces the risk of pathogen resistance development. In this study, our objective was to screen for actinomycetes that can effectively inhibit clubroot. We screened 13 actinomycete strains, identifying 2, XDS3-6 and CD1-1, with substantial in vivo inhibitory effects, achieving infection suppression rates above 64% against *P. brassicae*. Phylogenetic analysis classified XDS3-6 and CD1-1 as *Streptomyces virginiae* and *Streptomyces cinnamonensis*, respectively. Both strains exhibited protease and glucanase production capabilities, essential for pathogenic suppression. Additionally, these strains induced host defense responses, as evidenced by increased jasmonic acid (JA) and salicylic acid (SA) accumulation and elevated activities of defense-related enzymes. Colonization studies of XDS3-6 and CD1-1 mutant strains in cabbage roots indicated sustained root colonization, with peak colony-forming units (CFUs) at 20 days post-inoculation, reaching 11.0 × 10^4^ CFU/g and 8.5 × 10^4^ CFU/g, respectively, and persisting for at least 30 days. Overall, these findings underscore the potential of *Streptomyces* strains XDS3-6 and CD1-1 as effective biocontrol agents, providing a theoretical foundation for their application in managing clubroot in Chinese cabbage.

## 1. Introduction

Clubroot, a globally significant soil-borne disease, presents considerable economic impacts, primarily resulting from the infection of *Plasmodiophora brassicae*. This pathogen predominantly infects economically valuable crops within the *Brassicaceae*, including rapeseed, radish, mustard, and cabbage [1]. The pathogenic process involves spore reproduction, primarily through cortex infection, and can also involve asexual short-cycle processes [2]. Moreover, during the root hair infection stage, some secondary zoospores can stay in the soil, where they await the opportunity for subsequent root hair infection [3]. Therefore, clubroot is particularly difficult to be controlled.

Currently, the primary measures for controlling clubroot involve the application of chemical agents. However, excessive use of chemical pesticides can lead to severe environmental pollution and potential risks to human health [4]. Hence, the development of safe, effective, and environmentally friendly biocontrol agents holds significant importance for clubroot prevention and management [5]. Biocontrol bacteria exert their antibacterial effects through mechanisms such as parasitism, antibiosis, competition, and growth promotion. They also have the capacity to modulate plant physiological processes, promoting host plant growth and development [6]. Additionally, these bacteria can induce host resistance, bolstering the plant’s defense mechanisms against pathogens. For instance, Hu et al. demonstrated the effective biological control of Chinese cabbage clubroot by *Streptomyces alfalfae*, which also influenced the diversity of bacterial and fungal communities in Chinese cabbage rhizosphere while enhancing plant growth in greenhouse conditions [7]. Samiah et al. isolated and screened eight bacterial strains from cabbage rhizosphere soil, showing their ability to inhibit the germination of dormant *P. brassicae* spores [8].

Biocontrol bacteria also produce secondary metabolites, including alkaloids, antibiotics, siderophores, and hydrolytic enzymes, to hinder the growth of pathogenic bacteria [9]. These metabolites can disrupt the integrity of cell membrane structures by inhibiting protein and nucleic acid synthesis and related enzyme activities, altering the permeability of the cell membranes of pathogenic bacteria and thereby impeding their growth. Arie et al. reported the effective control of clubroot using *Phoma glomerata* JCM 9972, with its secondary metabolite aciloxetin demonstrating potent cabbage clubroot control. In addition, 250 μg/mL Epoxydon was found to completely inhibit the germination of dormant *P. brassicae* spores [10]. Li et al. showed that secondary metabolites produced by *Bacillus subtilis* XF-1, including funzimycin-like cyclic peptides, promoted the lysis of dormant spores [11]. Malik et al. isolated 37 chitin-decomposing bacteria, with strains TD9, TD11, TD15, and TD24 exhibiting strong antagonistic effects against *Rhizoctonia*, *Fusarium*, and *Anthracnose* in plate experiments [12]. Zhang et al. identified that lipopeptide compounds produced by *Bacillus velezensis* Jt84 disrupted the mycelium morphology of *Magnaporthe oryzae*, with iturin playing a pivotal role, significantly reducing rice blast incidence during the rice seedling stage [13]. In summary, biocontrol bacteria offer a promising and environmentally friendly approach for clubroot management, as they employ various mechanisms and secondary metabolites to combat pathogenic bacteria and enhance plant resistance. These findings underscore the potential of biocontrol agents in mitigating clubroot-related challenges.

Numerous studies have demonstrated that biocontrol bacteria and their fermentation broths can elicit alterations in plant defense enzymes, resistance-associated substances, and plant hormones, including phenylalanine ammonia-lyase (PAL), peroxidase (POD), polyphenol oxidase (PPO), salicylic acid (SA), jasmonic acid (JA), and ethylene (ET), among others, thus enhancing plant resistance mechanisms. Following pathogen infection, resistant plant varieties typically maintain elevated enzyme activities of PPO, POD, and PAL [14]. For instance, *Bacillus siamensis* LZ88 can induce POD and PPO to enhance tobacco resistance against brown spot disease of tobacco caused by *Alternaria alternata* [14]. Furthermore, biocontrol bacteria have been shown in numerous recent studies to boost POD, PPO, and PAL activities, as well as augment SA and JA content in plants, thereby fortifying plant disease resistance [15]. Biocontrol bacteria induce host resistance by promoting the expression of enzymatic activities (e.g., POD and PPO) in the host, thereby illuminating the pathway of their action mechanism.

In this research, we conducted antagonistic assessments involving 13 strains of actinomycetes, and we successfully identified two biocontrol bacterial strains that effectively inhibited *P. brassicae* infection in cabbage. The comprehensive analysis of their biocontrol efficacy, functional attributes, underlying mechanisms, and colonization within cabbage roots will establish a robust foundation for the study of biological management of cabbage clubroot.

## 2. Results

### 2.1. XDS3-6 and CD1-1 Can Effectively Inhibit P. brassicae Infection in Cabbage Roots

**Figure 1:**
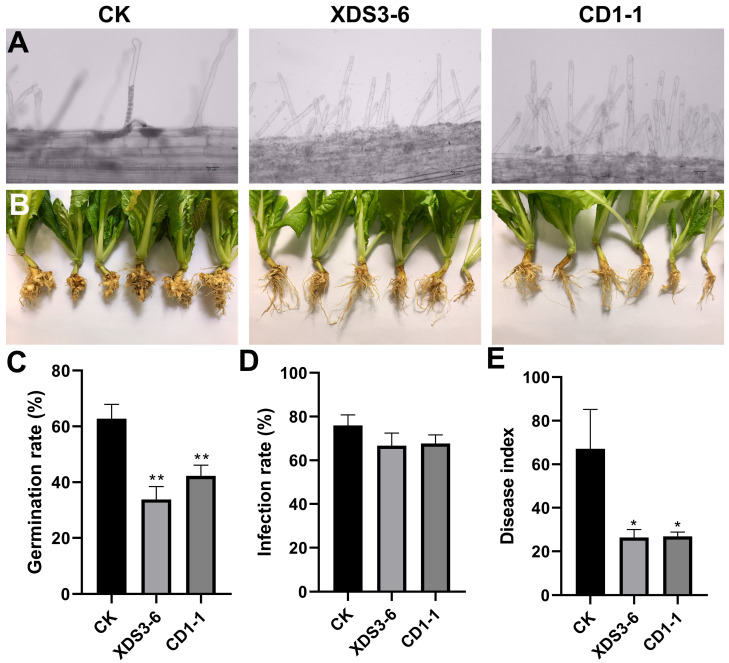
Biocontrol effects of XDS3-6 and CD1-1 on *P. brassicae*. (**A**) Phenotype of *P. brassicae* infecting root hair in Chinese cabbage treated with XDS3-6 and CD1-1 fermentation filtrate. (**B**) Phenotype of Chinese cabbage root on day 45 after inoculation with *P. brassicae* after treatment with XDS3-6 and CD1-1 fermentation filtrate. (**C**) Germination rate of dormant spores of *P. brassicae* treated with XDS3-6 and CD1-1 fermentation filtrate. (**D**) Root hair infection rate of *P. brassicae* treated with XDS3-6 and CD1-1 fermentation filtrate. (**E**) Root clubroot disease index of Chinese cabbage on the 45th day after inoculation with *P. brassicae* after treatment with XDS3-6 and CD1-1 fermentation filtrate. All experiments were repeated three times. Values represent means  ±  standard error from three replications. The statistical analyses were performed using Student’s *t*-test (* 0.01 < *p*  <  0.05, ** 0.001 < *p* < 0.01).

In our previous studies, a large number of actinomycete strains were isolated from soil ecosystems and exhibited varying degrees of antagonistic activity against *Monilinia fructicola* and *Phytophthora capsica* [6,16,17]. To further investigate their potential in controlling clubroot disease, we selected 13 representative strains for evaluation. Fermentation filtrates were prepared from these strains and subsequently tested for their effects on the germination of dormant spores and the root hair infection stage of *P*. *brassicae*. The results revealed that the fermentation filtrate of all 13 strains inhibited the germination of dormant spores of *P. brassicae* (Table S1). Contrastingly, a subset of strains—particularly LCD2-4, LCP4-2, CD2-7, ZAM1-9, and HCD3-7—consistently promoted root hair infection efficiency (Table S2). In contrast, the remaining strains demonstrated substantial inhibition of root hair infection by *P. brassicae*, with XDS3-6 and CD1-1 achieving inhibition rates of 41.8% and 32.64% on dormant spore germination and 12.25% and 10.94% on root hair infection, respectively (Tables S1 and S2 and Figure 1A,C,D). XDS3-6 was originally isolated from the rhizosphere soil near a border tree in Jinyun Mountain, Beibei District, Chongqing, while CD1-1 was isolated from grassland soil in Gele Mountain, Shapingba District, Chongqing. Subsequent in vivo assessments validated the efficacy of strains XDS3-6 and CD1-1 in managing clubroot. Under standardized greenhouse conditions (25 ± 1 °C, 70% RH), application of their fermentation filtrates resulted in disease suppression rates of 66.1% and 64.8%, respectively, demonstrating remarkable biocontrol efficacy against *P*. *brassicae* (Figure 1B,E). Furthermore, we conducted an evaluation of the broad-spectrum antifungal activity of XDS3-6 and CD1-1 using the plate confrontation method. Both strains exhibited significant broad-spectrum antifungal activity. CD1-1 effectively inhibited the growth of mycelia from various pathogens, including *Pestalotiopsis nicotiae*, *Phomopsis vexans*, *Botrytis cinerea*, *Colletotrichum nicotianae*, *Sclerotinia sclerotiorum*, *Thanatepephorus cucumeris*, *Phytophthora capsica*, and *Pythium delicense*, though with limited effect on *Rhizopus stolonifera*. Meanwhile, XDS3-6 inhibited the mycelium growth of seven pathogenic fungi, except *R. stolonifer* and *T. cucumeris*, achieving a mycelium growth inhibition rate exceeding 40% (Table 1). Collectively, these findings underscore the ability of strains XDS3-6 and CD1-1 to effectively suppress *P. brassicae* infection by targeting the inhibition of dormant spore germination and root hair infection, suggesting their potential as promising biocontrol agents against clubroot disease.

### 2.2. XDS3-6 and CD1-1 Both Belong to Streptomyces

**Figure 2:**
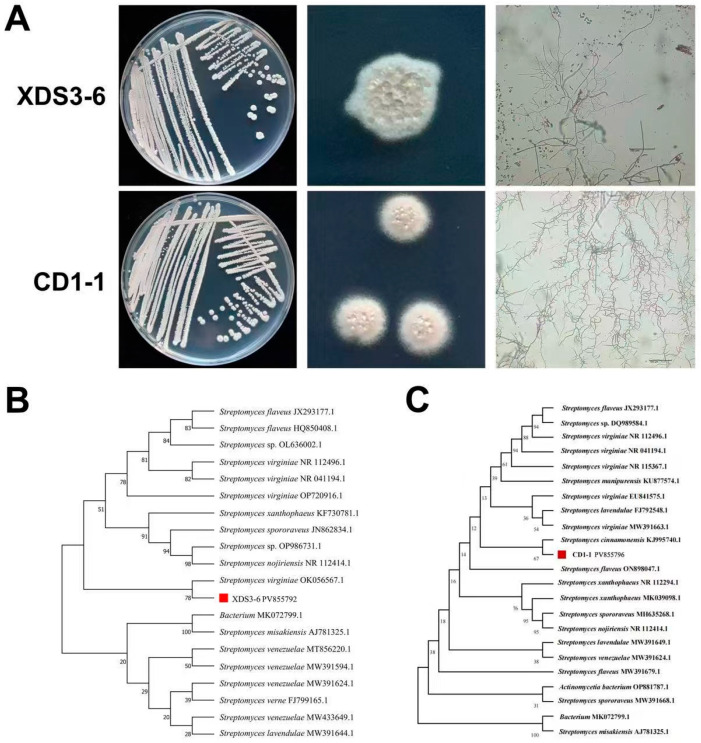
Identification of XDS3-6 and CD1-1. (**A**) Morphological characteristics of XDS3-6 and CD1-1. (**B**) Molecular biological identification of XDS3-6 based on *16s* rDNA using Neighbor-Joining phylogenetic tree. (**C**) Molecular biological identification of CD1-1 based on *16s* rDNA using Neighbor-Joining phylogenetic tree.

To confirm the taxonomic classification of strains XDS3-6 and CD1-1, we examined their growth patterns on Gauserime synthetic agar medium. Notably, no soluble pigments were produced by either strain. Based on optical microscopy, strain XDS3-6 exhibited spiral-shaped spore filament tips, characterized by long spore chains with numerous branches (Figure 2A). In contrast, strain CD1-1 formed round, single colonies with a pale pink hue and central water production. Similar to XDS3-6, CD1-1 did not produce soluble pigments. Microscopic analysis revealed long and straight spore filaments with multiple branches that were closely arranged in CD1-1 (Figure 2A).

To gain further insight into their taxonomic status, we conducted molecular analysis by amplifying a fragment of approximately 1400 bp using universal *16S* rDNA primers. Subsequently, the *16S* rDNA sequences of strains XDS3-6 and CD1-1 were subjected to a BLAST homology search in the NCBI database. The results indicated a high degree of sequence homology, with strain XDS3-6 (GenBank accession: PV855792) sharing 99.86% and 96.16% homology with *S. virginiae* strain T26 and *S. cinnamonensis* strain ZZ035, respectively. The Neighbor-Joining phylogenetic tree corroborated this finding, demonstrating that XDS3-6 clustered together with *S. virginiae* strain PB01 (Figure 2B). On the other hand, strain CD1-1 (GenBank accession: PV855796) exhibited 99.86% homology with *S. virginiae* strain THA-960 and *S. flaveus* strain X418. Similarly, the Neighbor-Joining phylogenetic tree showed that CD1-1 formed a distinct cluster with *S. cinnamonensis* strain ZZ043 (Figure 2C). Integrating the morphological characteristics observed on Gauserime synthetic agar medium with the results of molecular identification, we tentatively classified strain XDS3-6 as *S. virginiae* and strain CD1-1 as *S. cinnamonensis*.

### 2.3. XDS1-5 and CD1-1 Produce Different Hydrolases

**Figure 3:**
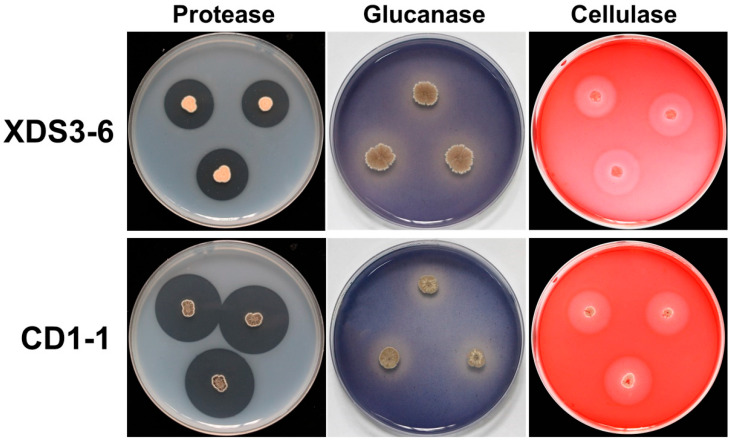
Protease, glucanase, and cellulase production characteristics of XDS3-6 and CD1-1.

The cell walls of *P. brassicae* resting spores are known to contain chitin, proteins, and polysaccharides, forming a thick and resistant structure that enables long-term survival in soil [18,19]. To investigate whether strains XDS3-6 and CD1-1 can impede the growth of *P. brassicae* through enzymatic hydrolysis, we conducted hydrolytic enzyme screening experiments. Both strains XDS3-6 and CD1-1 were inoculated onto hydrolase screening plates. Strain XDS3-6 exhibited the ability to produce protease, dextranase, and cellulase, but it did not demonstrate chitinase production (Figure 3). Among these enzymes, protease production was the most pronounced, resulting in a transparent circle with a radius of 8.20 mm. This was followed by glucanase activity, which yielded a transparent circle with a radius of 3.0 mm, while cellulase activity exhibited the weakest enzymatic activity, generating a transparent circle with a radius of 2.10 mm. In contrast, strain CD1-1 displayed the capacity to produce glucanase while lacking cellulase, dextranase, and chitinase production. Particularly noteworthy was CD1-1’s substantial protease activity, which resulted in a transparent circle with a radius of 25 mm. This radius was three times larger than that observed for strain XDS3-6 (Table 2).

### 2.4. XDS1-5 and CD1-1 Induce Resistance-Related Enzyme Activity and Reduce MDA Damage

**Figure 4:**
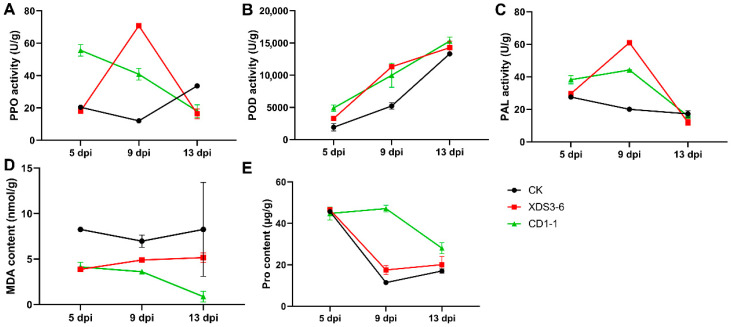
Effects of XDS3-6 and CD1-1 fermentation filtrate treatment on resistance-related enzyme activities in cabbage roots. (**A**) Activity of PPO after XDS3-6 and CD1-1 fermentation filtrate treatment in cabbage roots. (**B**) Activity of POD after XDS3-6 and CD1-1 fermentation filtrate treatment in cabbage roots. (**C**) Activity of PAL after XDS3-6 and CD1-1 fermentation filtrate treatment in cabbage roots. (**D**) Activity of MDA after XDS3-6 and CD1-1 fermentation filtrate treatment in cabbage roots. (**E**) Activity of Pro after XDS3-6 and CD1-1 fermentation filtrate treatment in cabbage roots.

POD, PPO, and PAL are vital defense enzymes in plants. Under stress conditions, plants autonomously adjust their defense responses to produce antibacterial substances. To investigate whether the fermentation filtrate of strains CD1-1 and XDS3-6 can influence the activity of these defense enzymes in cabbage roots and enhance cabbage root resistance to clubroot, we measured the activity changes of POD, PPO, and PAL. Following treatment with the fermentation filtrates of XDS3-6 and CD1-1, the activity of PAL, PPO, and POD in cabbage roots exhibited notable variations. In the CD1-1 fermentation filtrate treatment group, the activity of PAL, PPO, and POD was higher than that of the control group from 5 to 9 dpi, with the activity at 9 dpi being 2.2 times, 3.4 times, and 1.9 times that of the control group, respectively (Figure 4A–C). In contrast, the XDS3-6 treatment group displayed slightly lower PPO activity than the control group at 5 dpi (Figure 4A), while PAL and POD activities were slightly higher than the control group at 5 dpi and significantly higher at 9 dpi, reaching 3 times, 2.2 times, and 5.9 times that of the control group, respectively (Figure 4B,C). POD activity increased over time, with higher activity observed in the treatment group than in the control group. PAL and PPO activities were higher than those of the control group at 5–9 dpi but lower at 13 dpi, indicating that the two fermentation filtrates can increase POD activity within a specific timeframe, slowing the decrease in PAL and PPO activities, ultimately enhancing Chinese cabbage resistance (Figure 4A–C).

Pro serves a critical role in plant osmotic regulation and stress signaling pathways, while MDA, as a key product of membrane lipid peroxidation, exacerbates membrane damage. Research has demonstrated that the actinomycete *Streptomyces* sp. KLBMP5084 enhances Pro accumulation and reduces MDA levels in tomato seedlings, thereby facilitating their growth under salt stress conditions [20]. Proline (Pro) and malondialdehyde (MDA) content are indicative of plant resistance. Pro content in each treatment group was similar at 5 dpi, but from 9 to 13 dpi, Pro content significantly increased after XDS3-6 and CD1-1 treatments, with CD1-1 treatment resulting in the highest Pro content (Figure 4D). In contrast, MDA content in the treatment group was lower than that in the control group. At 13 dpi, MDA content in the CD1-1 treatment group was significantly lower than in the control group, representing just 1/13 of the control group. In particular, MDA content in cabbage roots remained relatively stable in the early stages of treatment but significantly decreased at 9 dpi, reaching 1/2.1 of the control group (Figure 4E). These results collectively demonstrate that each treatment regimen enhances the stress tolerance of cabbage by elevating its Pro content and reducing MDA levels.

### 2.5. XDS1-5 and CD1-1 Can Activate the Cabbage Hormone Pathway

**Figure 5:**
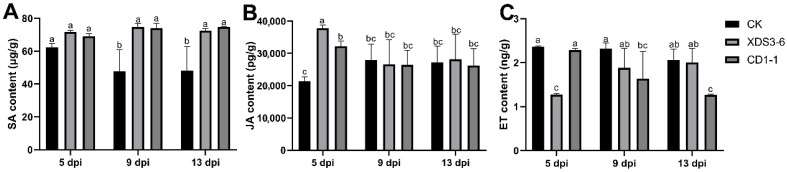
Effects of XDS3-6 and CD1-1 fermentation filtrate treatment on hormone content in cabbage roots. (**A**) Activity of SA after XDS3-6 and CD1-1 fermentation filtrate treatment in cabbage roots. (**B**) Activity of JA after XDS3-6 and CD1-1 fermentation filtrate treatment in cabbage roots. (**C**) Activity of ET after XDS3-6 and CD1-1 fermentation filtrate treatment in cabbage roots. All experiments we repeated three times. Values represent means  ±  standard error for three replications. The statistical analyses were performed using one-way ANOVA (Tukey’s HSD test). Lower case letters represent a significant difference (*p* < 0.05).

SA, JA, and ET serve as pivotal signaling molecules in plant defense responses, orchestrating stress response mechanisms and augmenting plant resistance. In this study, we sought to elucidate the dynamic alterations in ET, JA, and SA content within cabbage root tissues following treatment with strains CD1-1 and XDS3-6. The examination of ET content in the CD1-1 and XDS3-6 fermentation filtrate treatment groups revealed levels lower than those in the control group. Notably, the CD1-1 treatment group exhibited significantly reduced ET content compared to the control group at 13 dpi, representing a mere 1/1.62 of the control group’s content. Similarly, the ET content in the XDS3-6 treatment group was markedly lower than that in the control group at 5 dpi, with the control group’s content being 1.8 times higher than that of the treatment group (Figure 5A). Regarding JA content, both the CD1-1 and XDS3-6 fermentation filtrate treatment groups exhibited substantially elevated levels compared to the control group at 5 dpi, with content that was 2.0 times and 1.6 times that of the control group, respectively. Following this initial increase, the JA content in both treatment groups remained relatively stable and comparable to that of the control group (Figure 5B). In the case of SA, the CD1-1 and XDS3-6 treatment groups consistently maintained elevated contents. Specifically, at 9 dpi and 13 dpi, the SA levels were significantly higher than those of the control group, measuring at 1.55 times, 1.89 times, 1.56 times, and 1.51 times that of the control group, respectively (Figure 5C). These compelling findings underscore that XDS3-6 and CD1-1 systematically activate the SA and JA signaling pathways while concurrently inhibiting the ET signaling pathway, thereby initiating robust protection against clubroot in Chinese cabbage.

### 2.6. XDS1-5 and CD1-1 Can Colonize the Roots of Chinese Cabbage for a Long Time

**Figure 6:**
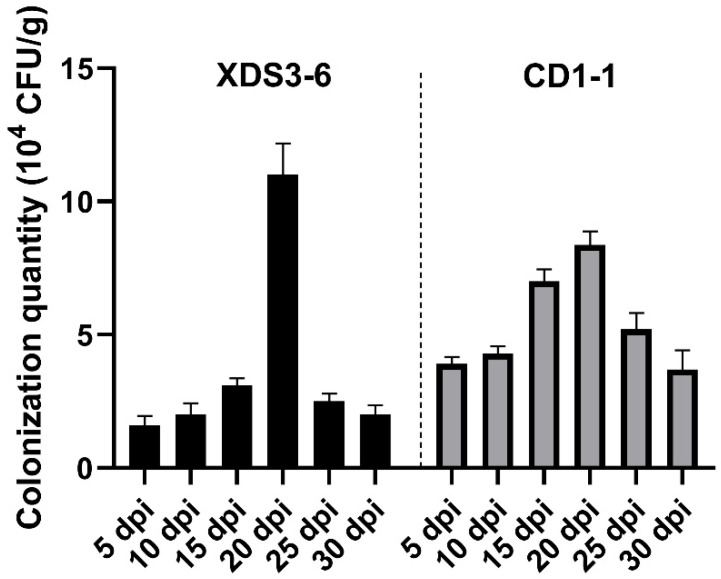
Colonization of XDS3-6 and CD1-1on Chinese cabbage roots. All experiments we repeated three times. Values represent means  ±  standard error for three replications.

In the field evaluation of biocontrol strains, it is of paramount importance to investigate not only the interactions between biocontrol strains, pathogens, and host plants but also their interactions with the soil and the environment. Equally critical is the ability of biocontrol strains to establish stable colonization within both the soil and the plants, as this profoundly influences their functional efficacy. To assess the colonization capability of strains XDS3-6 and CD1-1 within Chinese cabbage roots, we carried out rifampicin-resistance mutagenesis screening, resulting in the identification of two marker strains that exhibited robust growth on rifampicin plates containing 300 μg/mL rifampicin. These marker strains displayed identical colony morphology and coloration to the original strains. Subsequently, after ten generations of cultivation on rifampicin-free plates, the two marker strains were successfully transferred to plates with 300 μg/mL rifampicin, where they continued to thrive. This suggests that the rifampicin-resistant mutant strains CD1-1 and XDS3-6 selected by rifampicin exhibit excellent genetic stability, thereby laying a foundation for the quantitative assessment of their colonization within the host. Furthermore, both the XDS3-6 mutant strain and the CD1-1 mutant strain exhibited prolonged root colonization within cabbage plants, with colonization levels increasing over time. The peak colonization levels were achieved on the 20th day, measuring 11.0 × 10^4^ CFU/g and 8.5 × 10^4^ CFU/g, respectively. Subsequently, colonization gradually declined but maintained a consistent level compared to the preceding period (Figure 6). These findings provide strong evidence that strains XDS3-6 and CD1-1 can effectively suppress the development of clubroot by infecting and establishing colonization within Chinese cabbage roots.

## 3. Discussion

Clubroot, a disease characterized by its extensive distribution and rapid dissemination, poses a significant threat to Brassicaceae crops, leading to adverse effects on their growth, vitality, and economic value. However, the causative agent of clubroot obligately parasitizes the roots of living plants and cannot be cultured in vitro, making its prevention and treatment a formidable challenge [21]. Current strategies primarily involve breeding resistant plant varieties and resorting to chemical control methods [22]. However, these approaches often fall short in adapting to the evolving diversity of pathogen strains, raising concerns about safety, environmental pollution, and the emergence of fungicide-resistant strains associated with chemical control [23]. Embracing eco-friendly and sustainable biological control strategies can address these limitations, although their effectiveness typically lags behind chemical interventions, and stability can vary [24]. Moreover, the practical use of biological source pesticides in agriculture remains limited, so it is crucial to search for and develop biocontrol agents for the management of clubroot. For instance, *Phoma glomerata* JCM 9972, with its secondary metabolite aciloxetin, and *Bacillus subtilis* XF-1, including funzimycin-like cyclic peptides, can effectively promote the lysis of dormant spores of *P. brassicae* to reduce clubroot [10,11]. Thus, biocontrol offers a promising and environmentally friendly approach for clubroot management, as it employs various mechanisms and secondary metabolites to limit the growth and spread of pathogens and enhance plant resistance.

In our study, we identified two *Streptomyces* strains, XDS3-6 and CD1-1, that effectively suppressed root hair infections caused by *P. brassicae*, resulting in a remarkable 64% inhibition of cabbage clubroot development. To optimize our experiment, we introduced cabbage root exudates into the culture medium, adjusted pH levels to a weakly acidic range, and maintained a dark environment at 16 °C to enhance the germination rate of dormant spores. Within the hydroponic system, we standardized the dormant spore concentration at 10^7^ CFU/mL. Given that dark and weakly acidic conditions promote *P. brassicae* infection, our experimental design thoughtfully selected optimal culture conditions for infection assessment, which was performed on the 9th day [25]. Furthermore, we considered that excessively concentrated actinomycetes fermentation filtrate might harm hydroponic seedlings, while overly diluted concentrations might not affect the root hair infection process. These meticulous arrangements significantly enhanced the inhibitory effects of XDS3-6 and CD1-1 on clubroot development.

Notably, XDS3-6 and CD1-1 are classified as *Streptomyces* species, specifically identified as *S. virginiae* and *S. cinnamonensis*, respectively. Previous research has revealed the remarkable capabilities of *S. virginiae*, including its proficiency in transforming and degrading sterol compounds. Additionally, this strain produces Virginiamycin M (VM), a polyketide–peptide antibiotic hybrid, with the peptide antibiotic Virginiamycin S (VS) as its synergistic counterpart. VM and VS belong to the streptogramin family and exhibit potent synergistic antibacterial activity. Moreover, their secondary metabolism leads to the production of diverse novel sterol compounds, which exhibit inhibitory properties against a range of eukaryotic microorganisms [26,27,28]. In contrast, *S. cinnamonensis* can produce avidin-like biotin-binding proteins (streptavidins C1 and C2) that inhibit the mycelial growth of *Fusarium oxysporum* [29]. It is also capable of producing Monensin (a polyether ionophore antibiotic), which demonstrates remarkable anticoccidial and antitumor properties [30]. In sterile culture solutions, *Streptomyces xanthophaeus* strain T3-5 notably exhibits a substantial anti-rot effect on harvested strawberries, resulting in decreased titratable acidity and vitamin C content, as well as a delay in fruit firmness [31]. Our study extends the scope of biocontrol applications for these two *Streptomyces* species and elucidates their efficacy in inhibiting *P. brassicae*.

Given the structural similarities between the dormant sporangia wall of *P. brassicae* and fungal cell walls, our experiment focused primarily on selecting fungal pathogens for assessing the antifungal spectrum [32]. Thus, we chose the major components of fungal cell walls—namely proteins, glucans, cellulose, and chitin—as indicators to evaluate the ability of actinomycete strains CD1-1 and XDS3-6 to secrete hydrolases capable of degrading these four components. The results demonstrated that strain XDS3-6 produced glucanase, protease, and cellulase, while strain CD1-1 produced protease and glucanase. Furthermore, this study revealed that strain CD1-1 displayed significantly higher protease production compared to strain XDS3-6—the transparent zone diameter generated by CD1-1 was 16.8 mm larger than that produced by XDS3-6. Moreover, during the broad-spectrum screening of antifungal activity against pathogenic fungi, strain CD1-1 demonstrated more potent antifungal effects than strain XDS3-6 against *P. nicotiae*, *P. vexans*, *S. sclerotiorum*, *T. cucumeris*, and *P. capsici*. These results suggest that the inhibitory rates of strains XDS3-6 and CD1-1 on pathogenic fungal growth could be associated with protease activity. These findings suggest that the inhibitory rates of strain XDS3-6 and strain CD1-1 on pathogenic fungi growth may be correlated with protease activity.

The ability of biocontrol strains to colonize plant roots plays a critical role in their capacity to exert stable effects and resist external environmental influences [33]. The use of antibiotic markers has gained significant research attention in this context [34,35]. For instance, rifampicin was employed as a marker to label the highly effective biocontrol strain *Zhihengliuella aestuarii* B18, which demonstrates inhibitory effects against *P. brassicae*. This marked strain could colonize the rhizosphere soil for a minimum of 45 days, with the highest bacterial colonization observed on the 10th day, and continued presence within plant roots on the 24th day [36]. These findings underscore the importance of colonization by biocontrol strains in managing *P. brassicae* infection in Chinese cabbage. The clubroot pathogen, *P. brassicae* initiates its infection process by first aggregating and adhering to cabbage roots before proceeding to invade through root hairs, resulting in swelling and differentiation into gall-like structures. This process adversely affects water and nutrient absorption by the roots [37]. When biocontrol actinomycetes (*Pseudomonas fluorescens* PCL1751) preemptively occupy plant root niches, they engage in competitive interactions with *Agrobacterium tumefaciens* for space and nutrients [35]. Consequently, this occupation significantly disrupts the invasion process and impact of *P. brassicae*, effectively preventing the infection of cabbage by *P. brassicae*. However, it should be noted that in this experiment, antibiotics were used to label strains CD1-1 and XDS3-6, and the two labeled strains that had colonized Chinese cabbage were recovered [35]. Consequently, we were unable to directly observe the specific localization of biocontrol strain colonization on the roots, nor could we directly observe changes in *P. brassicae* colonization following the application of actinomycetes [38]. Further experiments are warranted to substantiate the content of anti-*P. brassicae* strains that compete with *P. brassicae* for space and nutrients within plant roots.

In summary, our investigation identified 2 *Streptomyces* strains, XDS3-6 and CD1-1, from a pool of 13 tested strains, which exhibit remarkable efficacy in inhibiting Chinese cabbage clubroot disease. These strains possess a unique ability to colonize Chinese cabbage root systems, leading to potent inhibitory effects. These have several inhibitory mechanisms, including proteases that facilitate the degradation of *P. brassicae* cell walls, and they activate the host plant’s resistance defense mechanisms. This dual mechanism approach not only prevents clubroot disease but also enhances overall disease resistance in Chinese cabbage. These findings significantly contribute to the theoretical foundation for implementing biological control strategies to manage clubroot diseases in Chinese cabbage.

## 4. Materials and Methods

### 4.1. Materials and Growth Conditions

*P. brassicae* was collected from the swollen roots of cabbage in the Xiannv Mountain area of Chongqing and stored in a −20 °C freezer [39]. *P. nicotiae*, *P. vexans*, *B. cinerea*, *C. nicotianae*, *S. sclerotiorum*, *T. cucumeris*, *P. capsici*, *P. delicense*, and *R. stolonifer* (provided by the Southwest University) were maintained by our laboratory and used for evaluating the broad-spectrum resistance of XDS3-6 and CD1-1. 

Disinfected Chinese cabbage (*B. rapa* subsp. *Pekinensis*, ‘*Degao 536*’, a variety widely cultivated in Chongqing, purchased from an agricultural materials business department in Beibei District, Chongqing) seeds were sown on sterile wet filter paper and germinated at 25 °C in an incubator (Shanghai Yiheng Instruments Co., Ltd., Shanghai, China), for 3 days, then the seedlings were transferred to individual centrifuge tubes containing 2 mL of Hoagland nutrient solution (Shanghai Yuanye Biotechnology Co., Ltd., Shanghai, China). Subsequently, the seedings were incubated in a controlled environment with a temperature of 16 °C, a relative humidity of 70%, and a light cycle of 16 hours of illumination followed by 8 hours of darkness and optimal humidity for a duration of 7 days. After this incubation period, were gathered the root exudates from the seedlings and sterilized them using a bacterial filter.

### 4.2. Fermentation Filtrate

Initially, actinomycetes were introduced into Gauserime synthetic agar medium. Following a 5-day incubation period, two 8 mm actinomycetes cakes were generated and subsequently inoculated into 20 mL of Gauserime synthetic liquid medium. This liquid medium was cultured at 28 °C with agitation at 180 rpm for 7 days. Subsequently, the fermentation filtrate from the 13 strains was adjusted to a concentration of 1 × 10^7^ CFU/mL for use in the subsequent experiments [16].

### 4.3. Determination of Antifungal Activity

Initially, the broad-spectrum antifungal activity against nine pathogenic fungi, namely *P. nicotiae*, *P. vexans*, *B. cinerea*, *C. micotianae*, *S. sclerotiorum*, *T. cucumeris*, *P. capsici*, *P. delicense*, and *R. stolonifer*, was determined using the plate confrontation assay. To conduct this assay, four distinct actinomycetes strains were inoculated at four equidistant points positioned 25 mm away from the center of the Petri dish. Antagonistic actinomycetes were similarly inoculated on both sides, 25 mm from the central point, and two 30 mm straight lines were drawn to create a cross-shaped pattern. These cultures were then incubated for three days, with an 8 mm agar plug placed at the central point. For the control group, pathogenic fungi were inoculated at the center of the Petri dish without the addition of actinomycetes. Each experimental setup was replicated three times. The inhibition rate (%) was calculated using the following formula:

Inhibition rate (%) = (control colony diameter − treatment colony diameter)/control colony diameter × 100

This formula was used to determine the percentage of inhibition based on the difference in colony diameters between the control and treatment groups.

### 4.4. The Germination of Dormant Spores and Infection of Root Hair of P. brassicae

We prepared dormant spore suspensions of *P. brassicae* using a standardized method [39], adjusting the final suspension to a spore concentration of 1 × 10^7^ CFU/mL using a hemocytometer. Chinese cabbage root exudates served as the culture medium. In sterilized Erlenmeyer flasks, 5 mL of root exudates, 0.5 mL of dormant spore suspension, and 0.5 mL of strain fermentation filtrate were combined. These flasks were then cultured under dark conditions at 24 °C. A Gauserime synthetic liquid medium was used as a control, and each treatment was replicated three times. On the 5th day, dormant spore germination was observed under a microscope. The methodology for evaluating the germination of *P. brassicae* resting spores was optimized based on Wu’s protocol [40]. Spores were stained with 1% orcein (dissolved in 45% acetic acid) for 10–15 s. Non-germinated spores appeared colored under the microscope, while uncolored spores were considered to have germinated. The spore germination rate and germination inhibition rate were calculated.

The cabbage seeds were sown on sterile wet filter paper and germinated in an incubator at 25 °C for 3 days. The seedlings were then transplanted into 10 mL light-proof centrifuge tubes filled with Hoagland nutrient solution for hydroponic culture. After five days, dormant *P. brassicae* spores and fermentation filtrate were inoculated into the centrifuge tubes. The final concentration of dormant spores was 10^7^ CFU/mL. In the treatment group, the fermentation filtrate was added at a 1:30 (*v*/*v*) ratio to the Hoagland solution, while the control group received Hoagland nutrient solution only. After nine days of hydroponic cultivation, the seedling roots were washed with water, dyed with a fluorescent pink dyeing solution (Beijing Solarbio Science & Technology Co., Ltd., Bejing, China) for 30 min, excess dyeing solution was rinsed, and root hair infection was observed under a microscope. 

### 4.5. In Vivo Anti-Clubroot Test

We prepared fermentation filtrates of strains CD1-1 and XDS3-6 at a concentration of 10^7^ CFU/mL, as well as a suspension of *P. brassicae* resting spores at 2.5 × 10^8^ spores/mL. We irrigated the roots of Chinese cabbage plants at the four true-leaf stage (10–25 days) with the fermentation filtrates of the strain XDS3-6 and *P. brassicae* resting spore suspension, ensuring that each gram of soil contained 3 × 10^7^ resting spores of *P. brassicae*. Subsequently, 20 mL of XDS3-6 fermentation broth was applied every 7 days for a total of 5 applications. Disease control was established by inoculating dormant *P. brassicae* spore suspension and watering with Gauserime synthetic liquid medium. This was repeated for 20 cabbage plants in each treatment group. Disease incidence was assessed 50 days after inoculation, and data were recorded based on the 6-level grading standard established by Wu [40]. The treatment procedure for CD1-1 was identical to that of XDS3-6.

### 4.6. Identification of Actinomycetes

For the purpose of morphological identification, strains XDS3-6 and CD1-1 underwent a structured procedure. Initially, each strain was inoculated onto Gauserime synthetic agar medium using the streaking method. Subsequently, the plates were inverted and incubated at 28 °C for a period of 7 days, during which time the growth characteristics of actinomycetes, as well as the production of soluble pigments in both aerial mycelium and substrate mycelium, were meticulously observed and documented. Following this incubation period, pre-sterilized coverslips were gently inserted into each plate at a 30° angle, with three coverslips placed in each dish. The plates were once again inverted and incubated at 28 °C for an additional 7 days. Subsequently, the coverslips were retrieved, and any excess agar was carefully removed from their undersides. A small quantity of distilled water was applied to a glass slide, and the coverslips were positioned on the slide. Utilizing an optical microscope, the morphological features of the hyphae and spore chains of the biocontrol strains were scrutinized and duly recorded [41].

For molecular identification, the DNA of strains XDS3-6 and CD1-1 was extracted following the instructions provided by the Magen Bacterial DNA Extraction Kit. Fragment amplification was conducted using the 16S rDNA universal primers 27F (5′-AGAGTTTGATCCTGGCTCAG-3′) and 1492R (5′-TACGGCTACCTTGACGACTT-3′) (Synthesized by Sangon Biotech (Shanghai) Co., Ltd., Shanghai, China). The PCR amplification reaction mixture (50 μL) comprised 25 μL of 2× Taq Master Mix (Takara Biomedical Technology (Beijing) Co., Ltd., Beijing, China), 2 μL each of the upstream and downstream primers, 2 μL of DNA template, and 19 μL of ddH2O. The PCR amplification protocol included an initial denaturation step at 94 °C for 3 min, followed by 29 cycles of denaturation at 94 °C for 30 s, annealing at 55 °C for 30 s, extension at 72 °C for 2 min, and a final extension at 72 °C for 10 min. Subsequently, the PCR product was sent to Shanghai Sangong Co., Ltd. for sequencing. The resulting sequence data were then assembled and compared using SeqMan in order to construct a phylogenetic tree [42].

### 4.7. Identification of Hydrolase Produced by Antagonistic Actinomycetes

The actinomycetes CD1-1 and XDS3-6 were separately inoculated onto different culture media, including:

ABP medium (for glucanase screening): Comprising 5.0 g of Poria powder, 1.0 g of K_2_HPO_4_ (Sichuan Chuanheng Holdings Group Co., Ltd., Sichuan, China)_._, 0.1 g of aniline blue (Shanghai Aladdin Biochemical Technology Co., Ltd., Shanghai, China), 1.0 g of KNO_3_ (King Chemical (Shanghai) Co., Ltd., Shanghai, China), 1.0 g of NaCl (Shanghai Aladdin Biochemical Technology Co., Ltd., Shanghai, China), 0.5 g of MgSO_4_ (Sinopharm Group Chemical Reagent Co.,Ltd., Shanghai, China), 0.1 g of FeSO_4_ (Sinopharm Group Chemical Reagent Co.,Ltd., Shanghai, China), 20.0 g of agar (Sinopharm Group Chemical Reagent Co.,Ltd., Shanghai, China), and 1000 mL of distilled water.

Protease screening medium: Prepared by mixing Solution A (240 mL sterile water + 6.4 g skimmed milk powder (Guangzhou Saigouo biotech Co., Ltd), sterilized at 121 °C for 1 min) and Solution B (6.4 g agar + 240 mL sterile water, sterilized at 121 °C for 10 min) post-sterilization.

CMC-Na medium (for cellulase screening): Containing 1.0 g of K_2_HPO_4_, 0.5 g of MgSO_4_, 10.0 g of sodium carboxymethylcellulose (Shanghai Aladdin Biochemical Technology Co., Ltd., Shanghai, China), 0.01 g of FeSO_4_ (Sinopharm Group Chemical Reagent Co.,Ltd., Shanghai, China), 0.4 g of Congo Red (Shanghai Aladdin Biochemical Technology Co., Ltd., Shanghai, China), 0.5 g of NaCl, 1.0 g of KNO_3_, 20.0 g of agar, and 1000 mL of distilled water.

Chitin enzyme screening medium: Composed of 0.3 g of KH_2_PO_4_ (Sinopharm Group Chemical Reagent Co.,Ltd., Shanghai, China), 10.0 g of colloidal chitin (Shanghai Aladdin Biochemical Technology Co., Ltd., Shanghai, China), 0.7 g of K_2_HPO_4_, 0.02 g of FeSO_4_, 0.5 g of MgSO_4_, 1.0 g of KNO_3_, 20.0 g of agar, and 1000 mL of distilled water.

Subsequently, each culture plate was inverted and placed in a 28 °C incubator. After a 7-day incubation period, the growth of the respective strains was observed, and the radius of any transparent circles formed was recorded. The presence of a strain’s growth and the development of a transparent circle indicated the strain’s ability to produce relevant hydrolytic enzymes for digesting specific cell wall components. Generally, a larger transparent circle radius corresponded to a higher hydrolase activity exhibited by the strain.

### 4.8. Determination of Enzyme Activity in Chinese Cabbage Roots 

Chinese cabbage roots bearing the fourth true leaf (10–25 days) were subjected to irrigation with a fermentation filtrate of strains XDS3-6 and CD1-1, each at a concentration of 10^7^ CFU/mL, while Gauserime synthetic liquid medium served as the control. At 5, 9, and 13 days post-inoculation (dpi), three cabbage plants were sampled from both the treatment and control groups. The root systems were meticulously cleaned and dried, and fresh, clean root tissues were excised and promptly preserved at −80 °C in a freezer. Approximately 0.10 g of root tissue was weighed and subsequently homogenized in an ice bath with the addition of 1.00 mL of extraction solution. Centrifugation at 8000× *g*, 4 °C, for approximately 10 min was conducted to collect the supernatant, which was then maintained on ice for subsequent analysis.

Following the kit instructions, the activities of PAL, POD, PPO, and the contents of proline (Pro) and malondialdehyde (MDA) within the Chinese cabbage roots subjected to the fermentation filtrate of strains XDS3-6 and CD1-1 were quantified according to the operation manual (Beijing Solarbio Science & Technology Co., Ltd., POD: https://img.solarbio.com/images/202505/1748506976471166084.pdf (accessed on 10 July 2025). PPO: https://img.solarbio.com/pdf/9-SHSJH/1-ZW/BC0195.pdf (accessed on 10 July 2025). PAL: https://img.solarbio.com/pdf/9-SHSJH/1-ZW/BC0215.pdf (accessed on 10 July 2025)). Additionally, cabbage root samples were dispatched to Chongqing Bonuoheng Biotechnology Co., Ltd. (Chongqing, China), where the contents of SA, JA, and ET were determined for those cabbage roots treated with XDS3-6 and CD1-1 fermentation filtrate, respectively.

### 4.9. Determination of Hormone Content in Chinese Cabbage Roots

For JA determination, we ground 0.5 g of plant tissue using a grinding homogenizer, then we added 7.5 mL of a methanol/acetonitrile mixture (80:20, *v*/*v*) and vortexed to mix thoroughly. Subsequently, ultrasonic-assisted extraction was conducted. Following extraction, the resultant solution was filtered using filter paper and concentrated through a rotary evaporator. It was then adjusted to volume with methanol to produce the analyte sample. Subsequently, the prepared sample was introduced into the LC-MS system for analytical evaluation. The chromatographic column demonstrated effective separation of JA and other compounds. The mobile phase, comprising methanol and 0.1% formic acid, was employed to ensure the stability and ionization efficiency of JA. The injector facilitated the introduction of the sample into the column, while the mass spectrometer detected and quantified the separated JA.

To extract SA, approximately 0.5 g of fresh plant sample was ground to a fine powder using liquid nitrogen. Subsequently, 5 mL of isopropyl alcohol/hydrochloric acid buffer was added, and the mixture was agitated for 30 min at 4 °C. Following this, 10 mL of methylene chloride was introduced, and the reaction mixture was maintained at 4 °C for an additional 30 min. The ensuing solution was centrifuged at 4 °C and 13,000 rpm for 5 min, and the resultant organic phase, sensitive to light, was carefully separated. The separated organic phase was then subjected to nitrogen blow-drying, then reconstituted in 250 μL–500 μL of methanol containing 0.1% formic acid. The reconstituted solution was further processed by incubating it on a 0.45 μm microorganism pore filter membrane and subsequently analyzed using HPLC-MS/MS.

Gas chromatography was employed to determine ET content. Plant samples were placed into a Soxhlet extractor and subjected to extraction using a methanol and ether mixture (volume ratio: 2:1). Following extraction, the resultant extract was dried over anhydrous sodium sulfate, and the organic solvent was evaporated using a rotary evaporator to yield a concentrated extract. A silica gel-packed column, coated with polydimethylsiloxane, was connected, and the concentrated extract was passed through the glass column for purification. The purpose of this purification step was to eliminate impurities from the sample, thereby enhancing the accuracy and stability of gas chromatography analysis. Ultimately, the purified sample was injected into the gas chromatograph using a syringe for analysis. In the gas chromatogram, the retention times of the ethylene standard and sample peaks exhibited consistency, enabling the calculation of ethylene content in the sample by comparing the peak areas of the standard and sample peaks.

### 4.10. Determination of Actinomycetes Colonization in Cabbage Roots

The strains XDS3-6 and CD1-1 were inoculated onto Gauserime synthetic agar medium for rifampicin resistance mutagenesis screening. A series of rifampicin concentrations, specifically 5, 10, 20, 30, 40, 60, 90, 120, 150, 210, and 300 μg/mL, were successively employed in sequential cultures until cells capable of thriving at the highest concentration of 300 μg/mL rifampicin were identified. These cells constituted a stable marker strain, displaying minimal alterations in colony morphology on the agar plates. Subsequently, the marker strain obtained from the screening process was cultured on Gauserime synthetic agar medium devoid of rifampicin. After undergoing ten generations of transfer, the marker strain was streaked onto agar plates containing 300 μg/mL rifampicin to assess its growth ability and confirm genetic stability.

Following this, actinomycetes from the marked strain were inoculated into Gauserime synthetic liquid medium without rifampicin. The culture was maintained at 28 °C with agitation at 180 rpm for 7 days, resulting in an actinomycete fermentation filtrate with a concentration of 10^7^ CFU/5 mL. This broth was then used to irrigate the roots of Chinese cabbage, specifically those at the fourth true leaf stage. Root tissue samples were collected at intervals of the 5th, 10th, 15th, 20th, 25th, and 30th days post-irrigation. Soil from the root surface was removed from the collected samples, followed by drying, and the marked actinomycetes present within the cabbage roots were isolated. To initiate the isolation process, cabbage root tissues were disinfected, involving a sequence of steps: 3 min of disinfection with 75% alcohol, followed by 3 min with 2% sodium hypochlorite, and a final 1-min disinfection with 75% alcohol. Subsequently, the tissues were rinsed three times with sterile water, and the sterile water used in the final rinse was examined to ensure thorough disinfection. Approximately 0.1 g of root tissue was then transferred to a sterile mortar, where it was homogenized with 5 mL of sterile water and a small quantity of quartz sand. After a brief period of standing, 100 μL of supernatant was collected and spread onto agar plates containing 300 μg/mL Rif. These plates were inverted and cultured at 28 °C. After 7 days, the presence or absence of growth of the labeled strain was assessed.

### 4.11. Statistical Analysis 

All experiments with corresponding data were repeated at least three times. The data are presented as means and standard deviations. The statistical analysis was performed with SPSS software (version 23.0) using Student’s *t*-test (* 0.01< *p*  <  0.05, ** 0.001 < *p* < 0.01, *** *p*  < 0.001) and one-way ANOVA (Tukey’s HSD test, *p* <  0.05).

## Figures and Tables

**Table 1:** The in vitro growth inhibition rates of nine pathogenic fungi by CD1-1 and XDS3-6.

Target Pathogen	Inhibition Rate (%)	
	CD1-1	XDS3-6
*Pestalotiopsis nicotiae*	44.99 ± 1.45 de	43.07 ± 1.24 d
*Rhizopus stolonifer*	0	0
*Phomopsis vexans*	49.27 ± 2.78 bcd	48.69 ± 1.82 c
*Botrytis cinerea*	59.02 ± 2.98 a	71.43 ± 1.86 a
*Colletotrichum micotianae*	47.09 ± 2.78 cde	65.33 ± 1.53 b
*Sclerotinia sclerotiorum*	49.29 ± 2.52 bcd	44.29 ± 1.5 cd
*Thanatepephorus cucumeris*	53.65 ± 1.44 abc	0
*Phytophthora capsici*	55.64 ± 1.57 ab	47.44 ± 1.56 cd
*Pythium deliense*	41.03 ± 0.74 e	48.72 ± 1.41 c

Note: All experiments were repeated three times with similar results. Values represent means  ±  standard error (SE) from three replications. The statistical analyses were performed using one-way ANOVA (Tukey’s HSD test, *p* <  0.05). The letters (e.g., a, b) are used to denote distinct groups, where each letter corresponds to a specific group. The significance of intergroup differences can be determined by comparing these letters: groups sharing the same letter indicate no statistically significant difference between them, whereas groups labeled with different letters signify a statistically significant difference.

**Table 2:** Summary of transparent circle width of protease, glucanase, cellulase, and chitinase produced by XDS3-6 and CD1-1.

Strain	Protease (mm)	Glucanase (mm)	Cellulase (mm)	Chitinase (mm)
XDS3-6	8.20 ± 0.06 b	3.00 ± 0.10 d	2.10 ± 0.00 e	0
CD1-1	25.00 ± 0.15 a	2.00 ± 0.10 e	0	0

Note: All experiments were repeated three times with similar results. Values represent means  ±  standard error (SE) from three replications. The statistical analyses were performed using one-way ANOVA (Tukey’s HSD test, *p* <  0.05). Letters (e.g., a, b) are employed to designate distinct groups, with each letter corresponding to a specific group. The significance of intergroup differences is determined by comparing these letters: groups bearing the same letter indicate no statistically significant difference, while those labeled with different letters signify a statistically significant difference.

## Data Availability

The data will be made available upon request.

## Supplementary Materials

The following supporting information can be downloaded at: https://www.mdpi.com/article/10.3390/plants14142195/s1, Table S1: Inhibiting effects of antagonistic actinomycete’s fermentation filtrate on germination of resting spores. Table S2: Inhibiting effects of antagonistic actinomycete’s fermentation filtrate on root hair infection.
