# Supplementary material for: Harnessing Streptomyces for the Management of Clubroot Disease of Chinese Cabbage (Brassica rapa subsp. Pekinensis)
Source: Plants (Basel). 2025 Jul 16;14(14):2195. doi: 10.3390/plants14142195 (PMC12300945; doi:10.3390/plants14142195)
Supplement: Supplementary file 1 [file plants-14-02195-s001.zip › plants-3693734-supplementary.pdf]

SUPPLEMENTARY INFORMATION

Harnessing *Streptomyces* for the Management of Clubroot Disease of Chinese Cabbage (*Brassica rapa* subsp. *Pekinensis*)

Shan Chen [1,†], Yang Zheng [2,†], Qing Wang [1,3], Rong Mu [1], Xianchao Sun [1], Guanhua Ma [1], Liezhao Liu [4], Jiequn Ren [2], Kuo Huang [5,*] and Guokang Chen [1,*]

1 Chongqing Key Laboratory of Plant Disease Biology, College of Plant Protection, Southwest University, Chongqing 400716, China; chen15807436519@163.com (S.C.); 13656607423@163.com (Q.W.); murong3430@163.com (R.M.); sunxianchao@163.com (X.S.); nikemgh@swu.edu.cn (G.M.)

2 Institute of Vegetable and Flower Research, Chongqing Academy of Agricultural Sciences, Chongqing 400055, China; renjiequn@outlook.com (J.R.)

3 Zhejiang Jiashan Forestry Technology Extension Station, Jiashan 314199, China

4 College of Agronomy and Biotechnology, Academy of Agricultural Sciences, Southwest University, Chongqing 400715, China; liezhao@swu.edu.cn

5 College of Food Science and Technology, Huazhong Agricultural University, Wuhan 430070, China

* Correspondence: hkztri@163.com (K.H.); chenguokang@swu.edu.cn (G.C.)

† These authors contributed equally to this work.

Table S1 Inhibiting effects of antagonistic actinomycete's fermentation filtrate on germination of *P. brassicae*

Strain	Germination rate（%）	Inhibited germination rate（%）
CC2-6	30.77 ± 3.74f	51.01 ± 5.95a
XDS3-6	33.80 ± 2.68ef	46.18 ± 4.26ab
TY2-4	34.87 ± 3.37def	44.48 ± 5.38abc
LCD2-4	36.50 ± 2.10cdef	41.88 ± 3.34abcd
HCD3-7	37.20 ± 2.62cdef	40.76 ± 4.18abcd
LCP4-2	38.90 ± 2.47bcde	38.06 ± 3.93bcde
CD1-1	42.30 ± 2.18bcd	32.64 ± 3.48cde
AMC3-4	42.53 ± 1.88bcd	32.27 ± 2.99de
ZAM1-9	44.00 ± 0.72bc	29.94 ± 1.15de
XDS1-5	44.97 ± 1.29b	28.40 ± 2.06e
LCD2-1	45.67 ± 1.36b	27.28 ± 2.16e
CD2-7	46.13 ± 1.37b	26.54 ± 2.18e
XDS2-4	56.90 ± 2.50a	9.39 ± 3.98f
CK	62.80 ± 2.93a	—

TableS2 Inhibiting effects of antagonistic actinomycete's fermentation filtrate on root hair infection

Strain	Infection rate （%）	Inhibited Infection rate（%）
CC2-6	17.17 ± 2.87e	77.41 ± 3.77a
LCD2-1	64.33 ± 2.11d	15.32 ± 2.78b
XDS3-6	66.67 ± 3.33d	12.25 ± 4.39b
AMC3-4	66.67 ± 3.33d	12.25 ± 4.39b
CD1-1	67.67 ± 2.27d	10.93 ± 2.99b
XDS2-4	68.33 ± 4.41d	10.05 ± 5.80b
TY2-4	72.57 ± 3.93cd	4.48 ± 5.17bc
XDS1-5	73.90 ± 4.85bcd	2.73 ± 6.39bcd
CK	75.97 ± 2.77bcd	—
LCD2-4	76.63 ± 5.45bcd	-0.87 ± 7.17bcd
LCP4-2	82.30 ± 2.87abc	-8.33 ± 3.78cde
CD2-7	84.73 ± 6.00ab	-11.54 ± 7.91de
ZAM1-9	89.40 ± 1.80a	-17.68 ± 2.37e
HCD3-7	90.33 ± 2.83a	-18.91 ± 3.73e
